# Characterization of adult patients with X-linked hypophosphatemia at a specialized center in Buenos Aires, Argentina

**DOI:** 10.20945/2359-4292-2024-0023

**Published:** 2025-03-24

**Authors:** Evangelina Giacoia, Laura María Schiró, Tatiana Martínez, María Celeste Balonga, Luisa Plantalech

**Affiliations:** 1 Servicio de Endocrinología y Metabolismo, Hospital Nacional Profesor Alejandro Posadas, El Palomar, Provincia de Buenos Aires, Argentina; 2 Servicio de Endocrinología, Metabolismo y Medicina Nuclear, Hospital Italiano, Ciudad Autónoma de Buenos Aires, Argentina

**Keywords:** X-linked hypophosphatemia, health-related quality of life, fibroblast growth factor-23, treatment adherence and compliance

## Abstract

**Objective:**

The study objectives were to characterize adult patients with XLH treated at a referral center, assess their physical function and the impact of X-linked hypophosphatemia (XLH) on their quality of life, and estimate their adherence to conventional treatment.

**Subjects and methods:**

Observational, retrospective study of patients with XLH from a referral center in Argentina, based on demographic and clinical data, complementary methods, and validated questionnaires (WOMAC, PROMIS, and SF-36).

**Results:**

Sixteen patients (age: 40.3 ± 13.2 years; female: 87.2%) were included. All patients had clinical and/or radiological skeletal manifestations (lower limb malformations and/or pseudofractures). The prevalence of clinical fractures was 60%. Hearing loss was the most frequent extra skeletal finding (67%). The WOMAC score was 47.8 ± 26 (62.5% of patients had ≥ 40 points). The PROMIS score was 23-33 (43% of patients) and ≥ 34 in 14% of patients. Except for emotional function, the median scores of the SF-36 domains were below 50 points. Only 20% of patients had good adherence to conventional treatment.

**Conclusions:**

Adult patients with XLH have numerous unmet needs, with frequent bone and extraskeletal complications. Physical function and quality of life scores were poor. Adherence to conventional treatment was unsatisfactory. Long-term studies are required to characterize these patients and confirm the efficacy and safety of continuous treatment, such as anti-fibroblast growth factor-23 monoclonal antibodies.

## INTRODUCTION

X-linked hypophosphatemia (XLH) is the most frequent inherited defect of renal tubular phosphate transport in humans (^1^). The incidence of XLH has been estimated at 3.9 to 5 cases per 100,000 live births (^2^). This disease is caused by loss-of-function mutations of the phosphate-regulating endopeptidase gene *(PHEX),* leading to an excess of circulating fibroblast growth factor-23 (FGF-23), with renal loss of phosphate and reduced synthesis of 1,25-dihydroxyvitamin D (^3^). In children, XLH is associated with delayed growth, short stature, craniosynostosis, muscle weakness, deformities of weight-bearing limbs, and dental disease (^2^). Among adults, symptoms may include skeletal (pain, enthesopathy involving the anterior spinal ligament, early osteoarthritis, and pseudofractures) and extraskeletal manifestations (dental complications, fatigue, hearing loss, Arnold-Chiari malformation) (^2^). As a consequence, affected patients develop impaired mobility, chronic pain, lower quality of life, and loss of productivity (^4,5,6^). In adults, XLH is associated with a remarkable disease burden and several unmet needs (^6^). In addition, data about XLH characteristics and outcomes in Latin America are scarce.

In this study, our objectives were the following: (A) to describe the clinical characteristics of adult patients with XLH assisted in a referral center in Argentina; (B) to evaluate the patients’ physical function and the impact of XLH on their quality of life; and (C) to estimate their adherence to conventional treatment.

## SUBJECTS AND METHODS

### Study design and data collection

We performed a retrospective, observational study at *Hospital Nacional Profesor Alejandro Posadas,* a tertiary care referral center in Argentina. Medical records of patients with a confirmed genetic diagnosis of XLH were retrieved for demographic characteristics (current age, age at diagnosis of XLH, sex, height, weight, body mass index [BMI]), skeletal manifestations (fractures, pseudofractures, bone deformities, need for corrective orthopedic surgery), and laboratory blood tests. Data about renal ultrasonography and brain computed tomography (CT) scans, when available, were collected for screening of complications.

Physical function, stiffness, and pain were evaluated using the validated Western Ontario and McMaster Universities Arthritis Index (WOMAC) instrument, which ranges from 0 (best score) to 100 (worst score) (^7^). Scores in the Patient-Reported Outcome Measurement Information System (PROMIS, which ranges from 0 as the worst score to 45 as the best score) (^8^) and the Argentine-Spanish SF-36 Health Survey (^9^) were also calculated. The SF-36 survey is a validated scale that quantifies eight health domains, namely, physical functioning, physical role limitations, bodily pain, general health perceptions, energy/vitality, social functioning, emotional role limitations, and mental health (^10^).

Treatment adherence was defined as a score ≥ 80% based on the validated Spanish version of the Compliance Questionnaire on Rheumatology (CQR), a self-administered questionnaire that evaluates patient’s compliance with prescribed treatment.

### Statistical analysis

Baseline data were collected between March 2018 and July 2019. Available data were anonymized and tabulated in a Microsoft Excel spreadsheet. In light of the descriptive, observational nature of our study, sample size estimation was deemed unnecessary. Continuous variables were described with mean and standard deviation, or median and range, according to their distribution pattern. Percentages were calculated for categorical and dichotomous variables. Missing values were not imputed, and outliers detected by the Grubbs test were excluded. P values < 0.05 were considered statistically significant. The statistical tests were performed using IBM SPSS Statistics, version 20.0.0 (IBM Corp., Armonk, NY, USA).

### Ethical considerations

Written consents were obtained from all participants. The study was approved by the Ethics Committee of Hospital Posadas (reference 274, code LUP0SO/19; January 21st, 2020), in accordance with Argentine regulations on clinical research and in compliance with the latest version of the Declaration of Helsinki.

## RESULTS

### Clinical and complementary test data

Sixteen patients with a confirmed diagnosis of XLH were included in our analysis. Most of these patients were female, and all of them were overweight or obese. Detailed information is available in Table 1.

**Table 1:** Main demographic characteristics of the study cohort

n	16
Age, mean ± standard deviation - years	40.3 ± 13.2
Sex - %	Female: 87.2%
Stature, median (range) - cm	135 (122-155)
BMI, median (range) - kg/m^2^ (*)	31.8 (27.2-63.1)
Age at diagnosis, median (range) - years (**)	5(1-59)
Time from diagnosis, median (range) - years (**)	32 (1-49)

Abbreviation: BMI, body mass index. (*) Outliers were excluded. (**) Missing values were not imputed.

**Figure 1:**
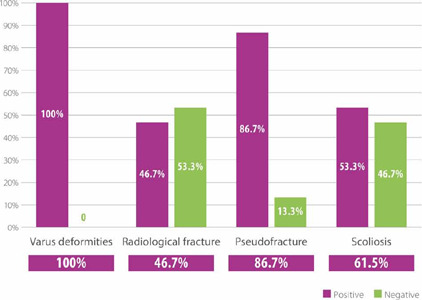
Radiological findings in 15 patients with available data.

Clinical fractures were found in 60% of the patients, with 25% reporting at least three fractures. All patients experienced skeletal deformities, and 68.75% (n = 11) reported having four deformities. Among participants with available surgical data, a median of 4 surgeries (range 2-8) were necessary for orthopedic correction. All patients had radiological findings, which are summarized in Figure 1. Serum and urine laboratory test results are shown in Table 2.

**Table 2:** Laboratory findings in the study cohort

Variable	Mean ± standard deviation	Normal range
**Serum**		
Total calcium	9.5 ± 0.4 mg/dL	8.6-10.2 mg/dL
Phosphorus	2.0 ± 0.3 mg/dL	2.5-4.5 mg/dL
Parathyroid hormone	86.8 ± 38.1 pg/mL	15-65 pg/mL
1,25-dihydroxyvitamin D	26.8 ± 11.9 ng/mL	>30 ng/mL
Bone alkaline phosphatase	37.9 ± 28.9 U/mL	5-26 U/mL
CrossLaps (*)	1587.7 ± 1138.3 pg/mL	<573 pg/mL
FGF-23 (*)	43.9 ± 26.9 pg/mL	≤134.04 pg/mL
**Urine (24 hours samples)**		
Phosphaturia (*)	665.7 ± 113.4 mg/24 h	
Calciuria	112.9 ± 60 mg/24 h	
Creatininuria (*)	927.9 ± 246.8 mg/24 h	
Calciuria/creatininuria (*)	0.14 ± 0.1	
Tubular reabsorption of phosphate (*)	76.1 ± 11.9%	
TmP/GFR	0.90 ± 0.36 mg/dL (**)	

Abbreviations: FGF-23, fibroblast growth factor-23; TmP/GFR, ratio of tubular maximum resorption of phosphate (TmP) to glomerular filtration rate (GFR). (*) Missing values were not imputed. (**) 25% of patients with available data were receiving conventional therapy.

### Extraskeletal complications

Data from renal ultrasonography were available for 13 patients. One patient had nephrocalcinosis, and two had nephrolithiasis. Ultrasonography showed no abnormal findings in 77% of our cohort. Brain CT scan data were available for eight patients, all of whom had normal results.

Hearing loss, as determined by audiometric tests and evaluated by an otorhinolaryngologist, was reported in 67% of the participants.

### Validated scores results

The mean (± standard deviation) total WOMAC score was 47.8 ± 26 points, with 62.5% of the patients ranking ≥ 40 points. The total PROMIS score, calculated based on available data from 14 participants, was 0-11 points in 7% of patients, 12-22 points in 36%, 23-33 points in 43°%, and ≥ 34 in 14°% of them.

**Figure 2:**
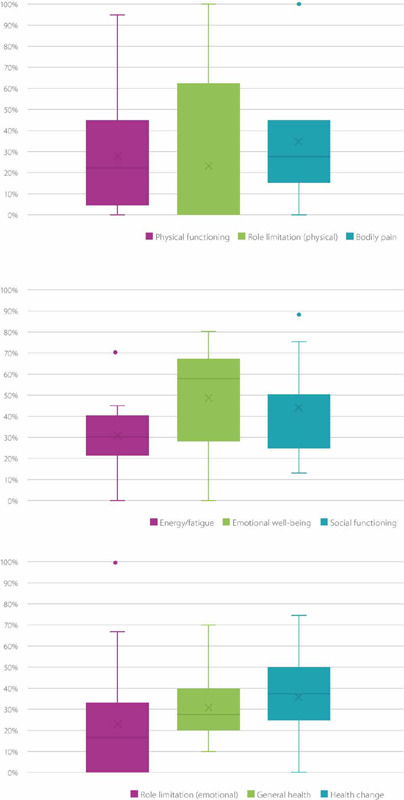
Scores in the SF-36 domains for the entire cohort. Boxes represent interquartile range. Lines represent range (minimum and maximum). Colored dots represent outliers. Central “x” represents the median score.

Except for the emotional role domain, which had a median score of 55 points (range 0-80 points), the median scores for all other individual domains of the SF-36 questionnaire were below 50 points (Figure 2).

### Adherence

According to data retrieved using the CQR tool, treatment adherence was good in 20% of the patients.

## DISCUSSION

In our cohort of adult patients with a confirmed diagnosis of XLH, all participants had clinical and/ or radiological skeletal complications and very low adherence to conventional treatment. As shown by SF-36 scores, the health burden of XLH was also remarkable.

The female predominance in our cohort may be related to the pattern of XLH inheritance. Affected males transmit the *PHEX* pathogenic variant to all their daughters, who will be heterozygotes and will be affected, but to none of their sons. By contrast, affected females have a 50% chance of transmitting the pathogenic variant to their offspring.

Notably, XLH is characterized by skeletal abnormalities of variable severity both in children and adults. Mutations in the *PHEX* gene are associated with increased secretion of FGF-23 by osteocytes, inducing phosphate wasting, hypophosphatemia, impaired endochondral ossification, and alterations of bone matrix and mineralization that persist for life (^11^). Skeletal deformities (including lower limb deformities) and radiological manifestations (including fractures and pseudofractures) were reported in all adult patients in our cohort, including clinical fractures in 60% of cases. In addition, all participants had radiological manifestations, including lower limb deformity (varus deformities) and pseudofractures (insufficiency fractures) which were present in 100% and 86.7% of the patients, respectively. In this population, the risk of fractures and pseudofractures in weight-bearing bones is associated with low bone turnover and does not usually depend on bone mineral density (^12^).

To prevent the development of pseudofractures and fractures, long periods of strenuous exercise (including walking or weight-bearing labor) should be avoided in patients with uncontrolled chronic hypophosphatemia (^12^).

Among our patients, hearing loss was the most frequent extraskeletal finding (67%). The prevalence of this complication in patients with XLH is highly variable and ranges from 16% to 76%; the exact pathogenesis of hearing loss is unclear, but bone malformation linked to osteomalacia and endolymphatic hydrops resulting from hypophosphatemia have been proposed as potential causes (^12,13^).

Managing the symptoms in patients with XLH requires a multidisciplinary approach to optimize their quality of life and reduce the burden of this chronic condition (^14^). In our cohort, with the exception of the emotional role, the median scores were low for all the remaining specific domains of the validated SF-36 tool (physical functioning, physical role limitations, bodily pain, general health perceptions, energy/vitality, social functioning, and mental health). These results show a poor quality of life in our XLH population, consistent with previous European research (^15,16^). It is worth noting that after transitioning to adolescence, the burden of XLH becomes multifactorial, with an increasing prevalence of anxiety and low self-esteem (^14^). Of note, WOMAC may represent the most suitable tool for clinical practice, taking into consideration that this self-administrated questionnaire may be easily completed at home and then sent back by email or by any smartphone application. Multidisciplinary interventions may be necessary to improve the quality of life and the general health of these patients.

Adherence to conventional treatment in our population was reportedly low. Adult symptomatic patients are typically prescribed vitamin D and oral phosphate salts to reduce osteomalacia and its consequences (^17^). Nevertheless, conventional treatment is difficult to implement, due to the frequent gastrointestinal adverse events and the need for frequent dosing (^3^). It has been reported that both dosing frequency (^18^) and adverse effects (^19^) are linked to poor treatment adherence, especially in patients with chronic diseases. Burosumab, a recombinant human IgG1 monoclonal antibody that targets FGF-23, is a novel XLH therapy with favorable tolerability that may be administered every 4 weeks (^20^), probably leading to better adherence rates in these patients.

The main limitations of our research include its unicentric, retrospective design and the failure to exclude a potential bias in data collection. However, several strengths are highlighted, including our relatively large sample size in the context of an infrequent disease, the use of validated tools, and the low proportion of missing data. In addition, ours is probably the largest descriptive cohort of adult patients with XLH in South America.

In conclusion, adult patients with XLH are a population with several unmet needs. Skeletal and extraskeletal complications are common, and adherence to conventional therapy is unsatisfactory. Further long-term research is warranted to characterize these patients and to confirm the effectiveness and safety of continuous treatment, with a special focus on anti-FGF-23 monoclonal antibodies in adults.
